# 

**Published:** 1871-09

**Authors:** 


					﻿Vol. XII.
SEPTEMBER, 18 71.
No. 9.
CHICAGO
Medical Examiner,
N. S. DAVIS, M.D., Editor,
AND
F. H. Davis, M.D., Assist.-Editor.
TERMS, S3.OO E’ER A-iVIVETAT, EV ADVANCE.
CHICAGO:
ROBERT FERGUS’ SONS, PRINTERS
12 CLARK AND 242—248 ILLINOIS STREET.
1871.
				

